# The role of *Akkermansia muciniphila* in maintaining health: a bibliometric study

**DOI:** 10.3389/fmed.2025.1484656

**Published:** 2025-02-03

**Authors:** Fangfang Gao, Canyu Cheng, Runwei Li, Zongcun Chen, Ke Tang, Guankui Du

**Affiliations:** ^1^Department of Breast Surgery, The First Affiliated Hospital of Hainan Medical University, Haikou, China; ^2^Key Laboratory of Tropical Translational Medicine of Ministry of Education, School of Basic Medicine and Life Sciences, Hainan Medical University, Haikou, China; ^3^Department of Endocrinology, The Second Affiliated Hospital of Hainan Medical University, Haikou, China; ^4^Department of Biochemistry and Molecular Biology, Hainan Medical University, Haikou, China

**Keywords:** *Akkermansia muciniphila*, bibliometrics, gut microbiota, gut-brain axis, metabolic disorder

## Abstract

**Background:**

*Akkermansia muciniphila*, as a probiotic, is negatively linked to IBD, obesity, and T2DM. The aim of this study was to comprehensively assess the research status of *Akkermansia muciniphila* over the past decade and explore the relationships between this bacterium and various health-related aspects.

**Methods:**

Tools VOSviewer, Bibliometrix, and CiteSpace were used to analyze various aspects including publication metrics, contributors, institutions, geography, journals, funding, and keywords.

**Results:**

Over the past decade, research on *Akkermansia muciniphila* has demonstrated a consistent annual growth in the number of publications, with a notable peak in 2021. China led in the number of publications, totaling 151, whereas the United States exhibited a higher centrality value. Among the 820 institutions involved in the research, the University of California (from the United States) and the Chinese Academy of Sciences (from China) occupied central positions. Willem M. De Vos ranked at the top, with 12 publications and 1,108 citations. The journal GUT, which had 5,125 citations and an Impact Factor of 23.0 in 2024, was the most highly cited. The most cited articles deepened the understanding of the bacterium’s impact on human health, spanning from basic research to translational medicine. Thirty-nine high-frequency keywords were grouped into five clusters, illustrating *Akkermansia muciniphila*’s associations with metabolic diseases, chronic kidney disease, the gut-brain axis, intestinal inflammation, and Bacteroidetes-Firmicutes shifts.

**Conclusion:**

Given *Akkermansia muciniphila*’s anti-inflammatory and gut-barrier-strengthening properties, it holds promise as a therapeutic for obesity, metabolic disorders, and inflammatory conditions. Therefore, future research should explore its potential further by conducting clinical trials, elucidating its mechanisms of action, and investigating its efficacy and safety in diverse patient populations.

## Introduction

In the intricate landscape of the gut microbiome, *Akkermansia muciniphila* stands out as a remarkable microorganism. Native to the gastrointestinal tracts of both humans and various animal species, it is celebrated for its distinctive ability to degrade mucins (1, 2). This bacterium was first identified by Derrien et al. (3) when they successfully cultured it using pure mucin as the sole carbon substrate, a milestone discovery that significantly advanced gut microbiome research. *Akkermansia muciniphila* is intricately involved in multiple aspects of maintaining gut health from early life. Appearing across different stages of human development, it serves as an important indicator of the vitality and biodiversity of the infant gut microbiota. Its abundance steadily increases from the neonatal period to adulthood (4).

Functionally, *Akkermansia muciniphila* plays a fundamental role in several key physiological processes within the gut. It is crucial for enhancing intestinal mucus secretion, maintaining the balance of mucosal viscosity, and safeguarding the integrity of the intestinal epithelial barrier (5–7). By degrading mucosal components, *Akkermansia muciniphila* generates short-chain fatty acids. These fatty acids play a dual role: they effectively reduce inflammatory reactions and prevent an increase in intestinal permeability (8, 9). Additionally, research has shown that it can increase the thickness of the intestinal lining, strengthen barrier functions, and promote the growth of beneficial microbial communities (10, 11). Maintaining gut homeostasis is of utmost importance as disruptions in intestinal physiology are closely linked to the development of various health issues, including metabolic syndromes, immunological disorders, infections, and neoplasias (12, 13). In this context, *Akkermansia muciniphila* emerges as a key player in preserving gut homeostasis.

Bibliometrics is a discipline that utilizes lexical analysis, citation analysis, and co-occurrence analysis to quantify document attributes and associated processes. Bibliometric analyses can yield valuable insights for guiding future research directions. The aim of this study is to conduct a bibliometric analysis of *Akkermansia muciniphila* bacteria and its relationship with health from 2013 to 2024, with the goal of identifying research hotspots and key topics for researchers in this field, and providing novel insights and research directions for future investigations.

## Methods

### Data source and literature search strategy

**Figure 1:**
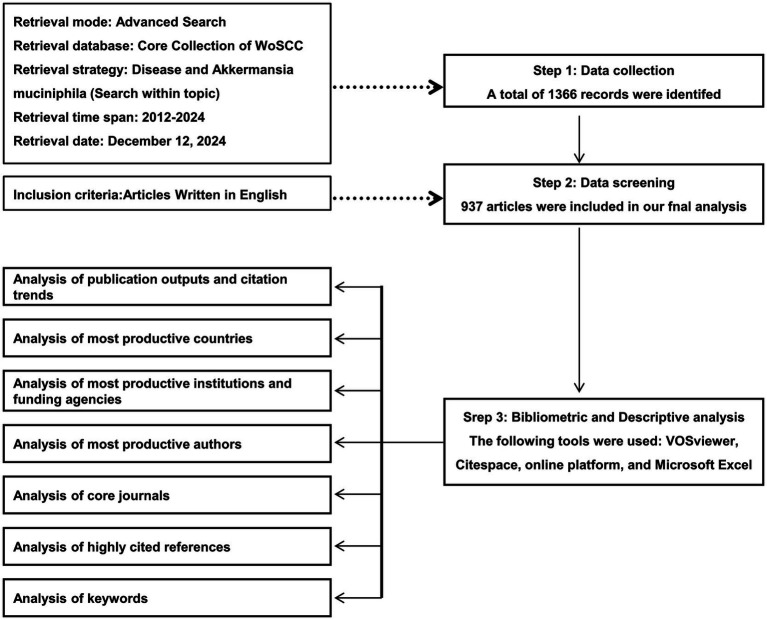
Flowchart of literature filtering and data analysis.

The Web of Science database was selected as the primary source for this study due to its extensive coverage of over 12,000 academic articles and its regular use by scholars. Compared to other databases such as Scopus, Medline, and PubMed, Web of Science provides the most comprehensive and reliable bibliometric analysis (6). On December 12, 2024, all versions of the database were utilized to search for and export relevant articles from the Web of Science (WoS) Core Collection. The complete records and cited references were then retrieved from the relevant publications and saved in plain text format for future analysis. By applying the following search parameters: disease (search within topic), article (document types), and TI = (“*Akkermansia muciniphila*”) OR AB = (“*Akkermansia muciniphila*”), 937 articles were obtained from the WOS database. A plain-text file was exported with all the full records and cited references for further analysis (Figure 1).

We considered all retrieved articles to characterize the different subject areas and document types of *Akkermansia* literature. For determining the share of each study design, targeted population, and conditions/diseases, we focused on original articles. To assess other bibliometric characteristics, we considered both original articles and reviews, including the year distribution of publications, citation numbers, most productive journals, institutions, sponsors, authors, and countries. Additionally, we examined the collaboration between keywords, terms, authors, and countries.

Subsequently, after employing clustering software to group the keywords into five distinct categories, a meticulous process of literature selection was carried out. Specifically, we sifted through a vast body of literature and ultimately identified 101 relevant documents associated with these categories. These selected 101 papers were then subjected to in-depth discussion and analysis with respect to *Akkermansia muciniphila*. Each of these literatures was carefully examined to extract valuable insights regarding various aspects of *Akkermansia muciniphila*, such as its physiological functions and potential applications in different fields. During the discussion phase, we compared and contrasted the findings presented in these papers, aiming to identify commonalities, discrepancies, and emerging trends. This comprehensive analysis enabled us to gain a more profound understanding of *Akkermansia muciniphila* within the context defined by the five keyword categories.

### Software for bibliometric analysis

Deduplication of the obtained data was performed using the CiteSpace (6.1.R1) (14). Two researchers independently extracted the publications, countries, institutions, funding agencies, authors, journals, citations, keywords, and highly cited references. To ensure data accuracy and reliability, discrepancies were reconciled via discussions and negotiations. Microsoft Excel was utilized to open the raw data in .xlxs format. The number of publications, grant money, and journals issued were then statistically compiled and analyzed. Bibliometric programs CiteSpace and VOSviewer (1.6.18) (15), run in a Java language environment, were employed to visualize the keywords, authors, and cited frequency of the literature, countries, issuing journals, issuing institutions, and issuing authors of the raw data from WOS in accordance with the corresponding format. BICOMB (2.02) (16), a tool for biomedical text mining and visualization, was used to extract and visualize medical-related data, such as gene-disease relationships and drug-disease associations, by importing Web of Science data. Cluster analysis was performed using the gCLUTO (1.0) (17) software’s biclustering algorithm, and 3D hill clustering graphs were generated by the multidimensional scaling method.

## Results

### Publication trends

**Figure 2:**
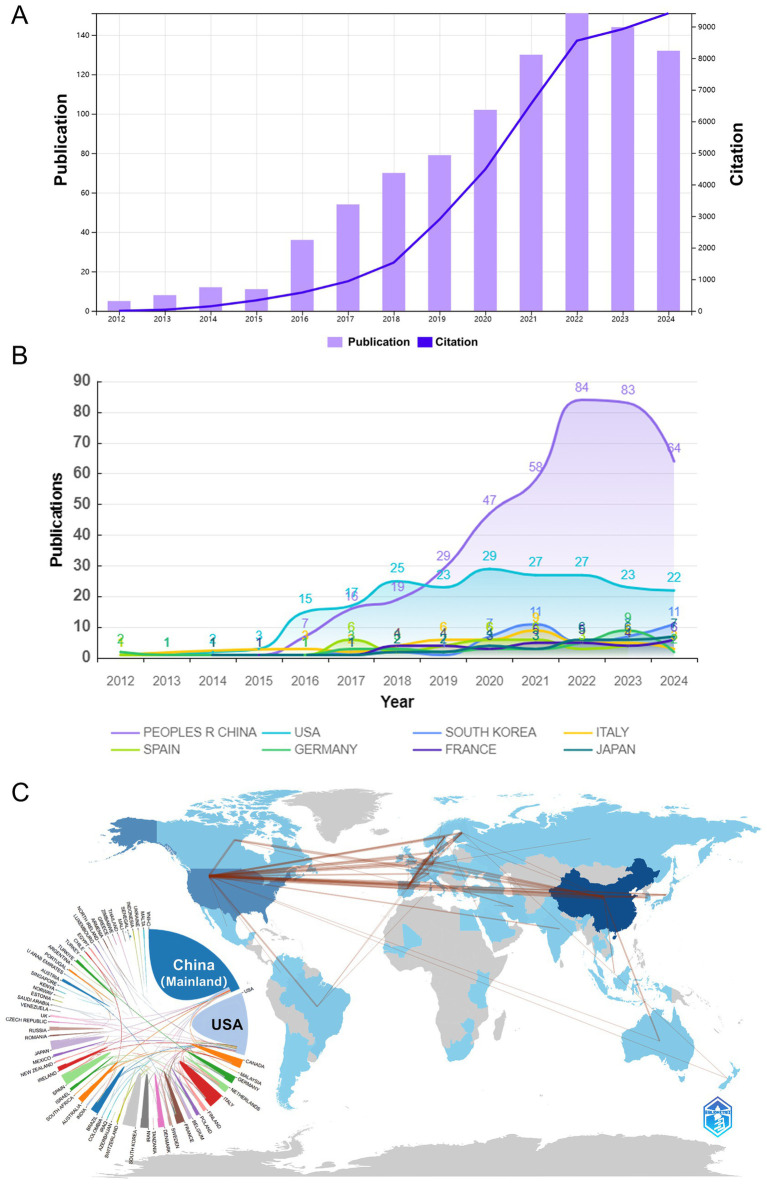
The publication trend of *Akkermansia muciniphila*. **(A)** Graph showing the number of publications per year over the past decade. **(B)** Chart displaying the number of publications issued by the top 10 countries from 2012 to 2024. **(C)** Map illustrating the cooperation of countries in the field of *Akkermansia muciniphila* from 2012 to 2024, along with the national and regional geographic distribution of *Akkermansia muciniphila*.

As of our search date in July 2024, the number of articles published in the field for that year may not be complete. Nevertheless, over the past decade, there has been a consistent annual increase in the number of articles published in the field, with a remarkable surge in 2021 (Figure 2A). Furthermore, Figure 2B illustrates a comparable trend in the predominance of the top 10 countries in the field.

### Analysis of country cooperation

In the last decade, research on *Akkermansia muciniphila* bacteria has been conducted by 52 countries and regions (Figure 2C). China has contributed the most with 151 publications, followed by the United States with 102 publications. The United States (USA) has a higher centrality value of 0.89, while China has a centrality value of 0.04.

### Analysis of institutional distribution

**Figure 3:**
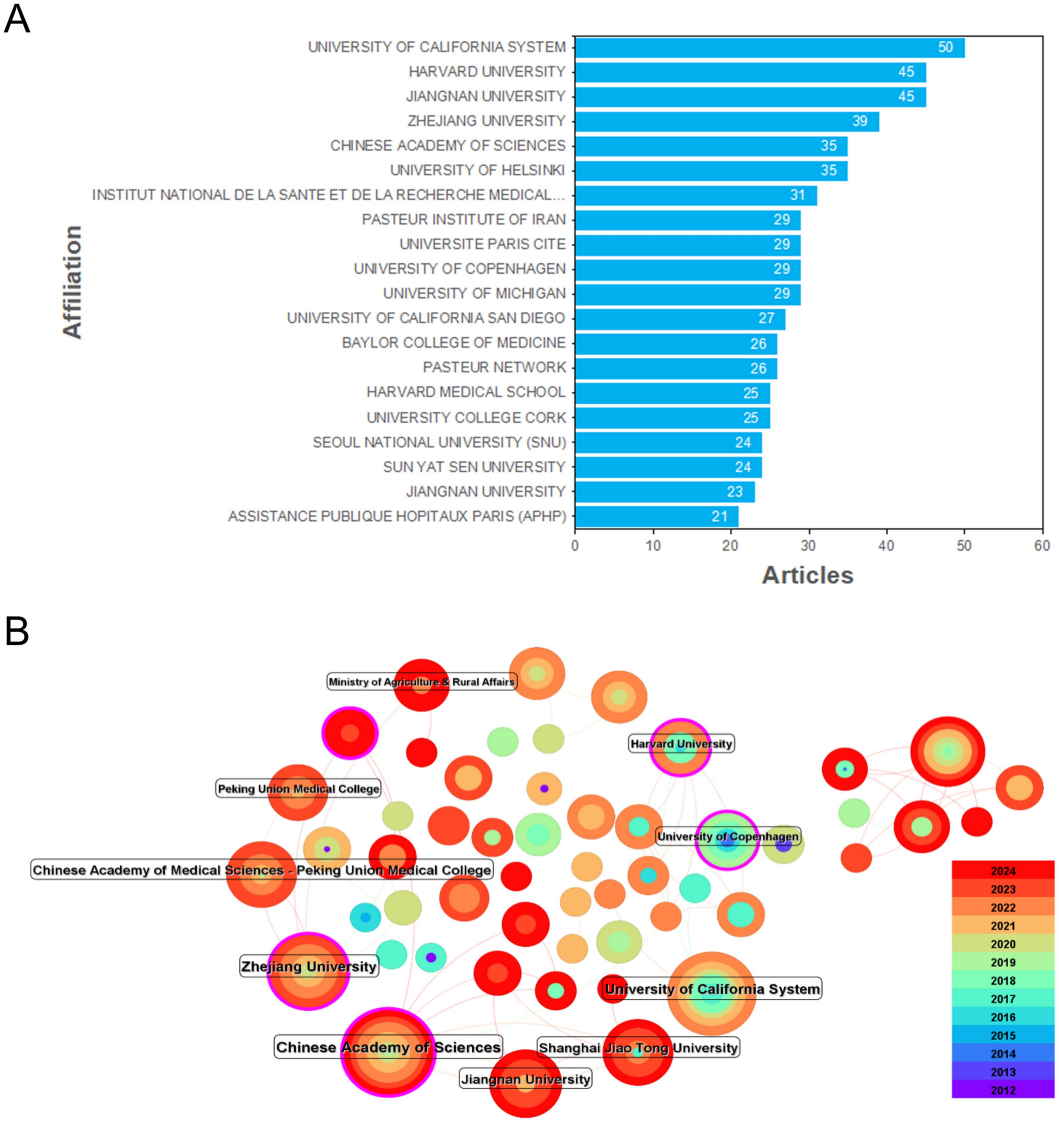
Information on institutional publications and collaborations. **(A)** The publication status of papers by various institutions. **(B)** Analysis of cooperation between institutions.

When analyzing the network hotspots of research institutions (Figure 3), a total of 820 institutions were found to be involved in this field, with 176 links. In terms of centrality, the University of California, Harvard University (both in the USA), and the Chinese Academy of Sciences, Zhejiang University, Jiangnan University, Peking Union Medical College, and Shanghai Jiao Tong University (all in China) have greater centrality values. The majority of the other universities in the top 10 are also from the USA and China. These institutions collaborate to varying degrees within their respective countries as a single unit, as well as with institutions in other countries and regions.

### Analysis of authors

Within the field focusing on the microbiota species *Akkermansia muciniphila*, our bibliometric assessment identifies a quintet of leading contributors whose works significantly shape the academic discourse (Table 1). Willem M. De Vos leads the ranking with a noteworthy publication count of 12 articles, garnering a cumulative citation tally of 1,108, which showcases his profound impact on *Akkermansia muciniphila* research. Hao Zhang closely follows with 11 contributions, having amassed 178 citations, reflecting a solid presence in the scientific community dedicated to studying this bacterial strain. Seyed Davar Siadat, also with 11 publications, secures his position with a total of 280 citations, substantially contributing to the knowledge base around *Akkermansia muciniphila*. Wei Chen, credited with 10 papers, has accumulated 158 citations, evidencing his active engagement in the exploration of this microbial area. Patrice D. Cani, despite sharing the same number of published articles as Chen, stands out with 2,693 citations, indicative of the high regard and broad influence his work receives in the scientific community. These authors collectively represent the forefront of *Akkermansia muciniphila*-centered investigations, and their combined efforts have substantially enriched the current body of knowledge and influenced future directions in this specialized microbiological domain.

**Table 1:** The top five authors in terms of *Akkermansia muciniphila* publications.

Rank	Author	Count	Total citations
1	De Vos, Willem M.	12	1,108
2	Zhang, Hao	11	178
3	Siadat, Seyed Davar	11	280
4	Chen, Wei	10	158
5	Cani, Patrice D.	10	2,693

### Analysis of most cited journals and papers

In identifying the most influential journals dedicated to disseminating *Akkermansia muciniphila* studies over the past decade, a comprehensive analysis reveals a roster of prestigious publications that have significantly contributed to advancing the scientific discourse surrounding this beneficial bacterium (Table 2). GUT stands as the leader in citations with a remarkable 5,125 tallies, holding an Impact Factor (2024) of 23.0, and is categorically placed within Gastroenterology & Hepatology. Its prominence reflects its role as a pivotal venue for cutting-edge research on the human gut ecosystem. Scientific Reports, with 2,106 citations, features an Impact Factor of 3.8 and spans Multidisciplinary Sciences, indicating its broad scope and interdisciplinary appeal in *Akkermansia muciniphila* studies. Frontiers in Microbiology, securing third place, has amassed 1,996 citations and carries an Impact Factor of 4.0, signaling its specialization in Microbiology and underscoring its dedication to bacterial and microbial sciences relevant to *Akkermansia muciniphila*. PLOS One, recognized for its focus on Multidisciplinary Sciences, has garnered 1,896 citations and an Impact Factor of 2.9 (2024), contributing to the rich tapestry of knowledge on the microbiome and health interrelations. Frontiers in Cellular and Infection Microbiology, positioned fifth, gathers 829 citations with an Impact Factor of 4.6, concentrating on Microbiology and reinforcing its stance as a prime forum for cellular and infectious diseases studies. These findings delineate the impact and breadth of *Akkermansia muciniphila* research across various scientific domains, showcasing how these top journals serve as epicenters for knowledge production and dissemination in this vibrant field.

**Table 2:** The top 10 most cited journals in the last decade.

Cited journal	Total citations	IF (2024)	WoS categories
Gut	5,125	23.0	Gastroenterology & Hepatology
Scientific Reports	2,106	3.8	Multidisciplinary Sciences
Frontiers in Microbiology	1,996	4.0	Microbiology
PLoS One	1,896	2.9	Multidisciplinary Sciences
Frontiers in Cellular and Infection Microbiology	829	4.6	Microbiology, Microbiology
Gut Microbes	662	12.2	Gastroenterology & Hepatology, Microbiology
Food and Function	572	5.1	Biochemistry & Molecular Biology, Food Science & Technology
Cell Reports	522	7.5	Cell Biology
Molecular Nutrition and Food Research	517	4.5	Food Science & Technology
Journal of Nutritional Biochemistry	516	4.8	Biochemistry & Molecular Biology, Nutrition & Dietetics

In mapping the intellectual landscape of *Akkermansia muciniphila* literature over the past decade, a review of the most cited articles reveals pivotal works that have significantly enriched the field through innovative perspectives and empirical evidence (Table 3). These studies stand as beacons in their respective areas, shaping theoretical frameworks and practical approaches toward understanding the multifaceted impacts of this bacterial genus on human health. These articles form the vanguard of *Akkermansia muciniphila* research, not only by virtue of their citation numbers but also due to their transformative impact on the discourse. Their collective influence spans diverse areas of inquiry, from basic biology to translational medicine, underlining the versatility and significance of *Akkermansia muciniphila* in human health and disease.

**Table 3:** The top five cited articles in the past 10 years.

Rank	Author	Year	Reference title	Journal	Cites
1	Cani P. D.	2018	Human gut microbiome: hopes, threats and promises	Gut	892
2	Anhê F. F.	2015	A polyphenol-rich cranberry extract protects from diet-induced obesity, insulin resistance and intestinal inflammation in association with increased *Akkermansia* spp. population in the gut microbiota of mice	Gut	879
3	Paone P.	2020	Mucus barrier, mucins and gut microbiota: the expected slimy partners?	Gut	802
4	Cekanaviciute E.	2017	Gut bacteria from multiple sclerosis patients modulate human T cells and exacerbate symptoms in mouse models	Proc Natl Acad Sci USA	659
5	Agus A.	2021	Gut microbiota-derived metabolites as central regulators in metabolic disorders	Gut	623

### Analysis of co-occurrence and cited keywords

**Figure 4:**
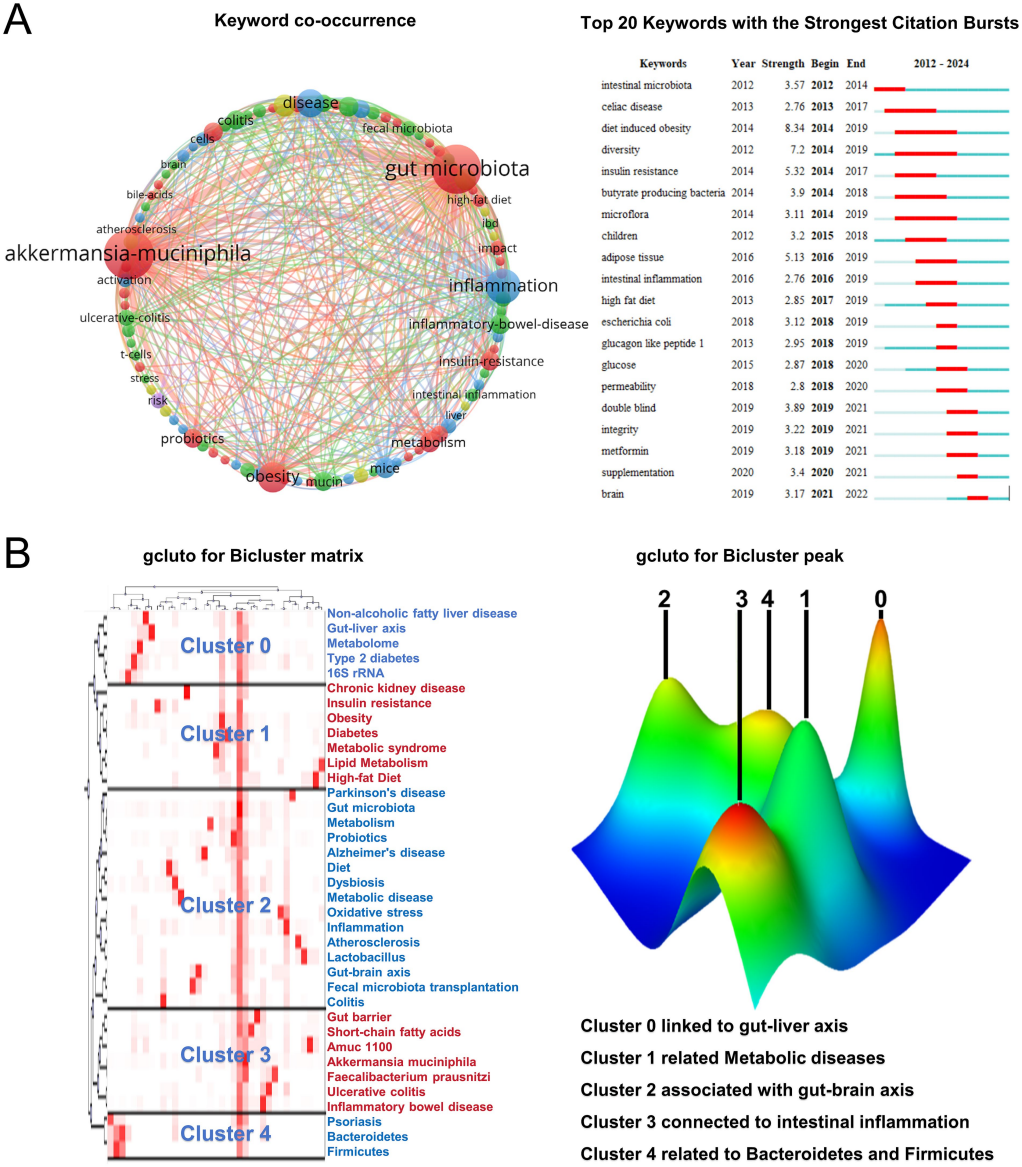
Keyword analysis. **(A)** Keyword co-occurrence diagram and the top 20 most cited keywords. **(B)** Bicluster matrix visualization of 31 high-frequency keywords and source articles, along with bicluster peak visualization of the same keywords and articles.

The term “gut microbiota” was the most frequently occurring term (179 occurrences), while “inflammation,” “obesity,” and “insulin resistance” also appeared multiple times (Figure 4A). To conduct biclustering analysis, high-frequency keywords with a frequency greater than or equal to 5 were selected (Figure 4B). A total of 39 eligible high-frequency keywords were identified and divided into five groups to construct a visualization matrix of high-frequency keywords and source literature. Cluster 0 suggests *Akkermansia muciniphila* has a relationship with metabolic diseases such as the gut-liver axis and NAFLD. Cluster 1 indicates a relationship between *Akkermansia muciniphila* bacteria and chronic kidney disease, insulin resistance, obesity, diabetes, metabolic syndrome, lipid metabolism, and a high-fat diet. Cluster 2 suggests a relationship with the gut-brain axis, Parkinson’s disease, and Alzheimer’s disease. Cluster 3 indicates a relationship between *Akkermansia muciniphila* bacteria and intestinal inflammation. Cluster 4 suggests a relationship with alterations in Bacteroidetes and Firmicutes. These findings provide valuable insights into the potential roles of *Akkermansia muciniphila* bacteria in various physiological processes and diseases.

## Discussion

Research on *Akkermansia muciniphila* has indeed witnessed rapid advancements. As documented in a bibliometric analysis from 2020, obesity (71 publications) and type 2 diabetes (39 publications) emerged as focal points of interest. More recently, in a 2022 bibliometric review, recurrent keywords highlighted the themes of oxidative stress, diet, metformin, fecal microbiota transplantation, short-chain fatty acids, polyphenols, and bacterial metabolites, underscoring multifaceted avenues of investigation. Our current study advances this body of knowledge by synthesizing subsequent developments and employing cluster analysis to elucidate connections. Specifically, we uncover *Akkermansia muciniphila*’s association with intestinal mucosal barrier function and its link to gut inflammation. Moreover, our findings reveal its relationship with inflammatory bowel disease, diabetes, obesity, non-alcoholic fatty liver disease and other metabolic disorders. Notably, *Akkermansia muciniphila* demonstrates intriguing ties to the gut-brain axis and neurological conditions, including Alzheimer’s disease, broadening its potential impact on health beyond traditional realms of metabolic syndromes. This comprehensive exploration underscores the evolving landscape of *Akkermansia muciniphila*’s physiological roles, illuminating its potential influences on both gut-centric and systemic health phenomena.

Within the scope of this study, our analytical framework hinged primarily on the Web of Science (WoS) database, where we meticulously evaluated research spanning from 2012 to 2024. The findings before 2022 echoed those delineated in previous bibliometric analyses, affirming the continuity and evolution of research trajectories within the field (18, 19). We complemented this core dataset with exhaustive examinations drawn from CNKI (a Chinese database), Scopus, and PubMed. This endeavor yielded 177 publications identified in CNKI, 897 in Scopus, and 605 in PubMed. Acknowledging the intrinsic variances across different data sources that may introduce systematic variations, dedicated analyses were conducted separately for each repository, as illustrated in Supplementary Figure S1. Across these databases, a remarkable coherence was observed, notably between Chinese and English repositories, indicating harmonized trends in topical directions. Despite navigational complexities posed by the multiplicity of databases, the consistent outcomes attest to the resilience and integrity of our discoveries.

High-frequency word analysis reveals that *Akkermansia muciniphila* influences the structure of the gut microbiota. Studies have shown that its abundance is often associated with a healthier gut environment, characterized by a diverse and balanced microbiota (20, 21). This bacterium’s role in maintaining gut barrier integrity and its anti-inflammatory properties contribute to the stabilization of the gut microbiome (22, 23). Research indicates that *Akkermansia muciniphila* can promote the growth of beneficial bacteria such as *Bifidobacterium* and *Lactobacillus* (24), while also influencing the levels of *Faecalibacterium prausnitzii*, another key species in gut health (25, 26). Moreover, *Akkermansia muciniphila*’s influence on the gut microbiome extends beyond direct effects on bacterial populations; it also affects the gut’s physical niche, such as mucin production, which can alter microbial community composition and functionality (27, 28). Therefore, *Akkermansia muciniphila* plays a critical role in shaping the gut microbiota’s diversity and structure of the gut microbiota, contributing to a balanced and healthy gut environment.

Furthermore, our analysis highlighted that *Akkermansia muciniphila* has an impact on gut microbiota metabolites. The metabolic activities of *Akkermansia muciniphila* not only directly affect the gut environment but also have indirect effects on the host’s systemic metabolism (29). *Akkermansia muciniphila*’s metabolism of mucin results in the production of short-chain fatty acids, particularly butyrate, which is a critical nutrient for colonic epithelial cells, promoting gut integrity and reducing inflammation (29–31). Through cross-feeding mechanisms, *Akkermansia muciniphila* influences the metabolism of other gut microbiota members, shaping the overall gut microbiome and metabolome (32). *Akkermansia muciniphila* can modulate tryptophan metabolism, an essential pathway with implications for gut health and beyond (33). The presence of *Akkermansia muciniphila* influences tryptophan derivatives, including indoleamine 2,3-dioxygenase 1 (IDO1) products, affecting gut-brain signaling and neuroprotection (34, 35). *Akkermansia muciniphila*’s impact extends to the host’s bile acid pool, where it can modify bile acid profiles, influencing host lipid and glucose metabolism (36, 37). These modifications in bile acid metabolism can help in the prevention of metabolic disorders, such as obesity and type 2 diabetes, by regulating the host’s energy homeostasis. Additionally, *Akkermansia muciniphila*’s influence on the production of gamma-aminobutyric acid (GABA), a neurotransmitter crucial for brain function, highlights its role in the gut-brain axis (31, 38). The modulation of GABA and other neurotransmitters by *Akkermansia muciniphila* may have implications for neurological conditions, such as depression and anxiety, suggesting potential therapeutic avenues (38, 39). Therefore, the metabolic activities of *Akkermansia muciniphila* play a crucial role in shaping the gut microbiome’s metabolic landscape by influencing short-chain fatty acids production, tryptophan metabolism, bile acid profiles, neurotransmitter levels, and other metabolic pathways.

*Akkermansia muciniphila*, particularly through its outer membrane protein Amuc_1100, has emerged as a versatile therapeutic agent with broad potential in promoting health and managing diseases (40). Amuc_1100 has demonstrated promise in stimulating GLP-1 secretion, improving metabolic health, and attenuating intestinal mucositis, thereby demonstrating potential in treating diabetes and chemotherapy-induced complications (41). Amuc_1100 has also been found to alleviate depression-like behavior in mice, suggesting benefits for mental health (42–45). Furthermore, its role in modulating CD8^+^ T cells to mitigate colitis-associated tumorigenesis and enhance immunotherapy efficacy highlights their potential in cancer treatment (46–48). Surface-displayed Amuc_1100 on lactic acid bacteria has shown potential in improving hepatic steatosis and gut health, offering a novel delivery platform (49). With its ability to regulate immune responses, gut barrier function, and metabolism (50–52), *Akkermansia muciniphila* and Amuc_1100 are promising candidates for novel interventions in a range of diseases, from metabolic disorders to neuropsychiatric conditions.

### *Akkermansia muciniphila* serve a crucial function in maintaining the barrier

*Akkermansia muciniphila* plays a crucial role in maintaining the integrity of the gut barrier. Recent studies have highlighted its influence on barrier function through various mechanisms. In alcoholic steatohepatitis, conventional type 1 dendritic cells preserve gut barrier integrity by sustaining populations of *Akkermansia muciniphila* (53). *Akkermansia muciniphila* upregulates genes critical for the intestinal barrier via activation of the ALPK1/TIFA pathway, which is dependent on ADP-heptose (54). Binding by intelectin-1 alters the localization of *Akkermansia muciniphila*, impacting mucus barrier modification (55). Grape polyphenols have been shown to positively influence *Akkermansia muciniphila* and enhance the gut barrier (56). Pasteurized *Akkermansia muciniphila* mitigates LPS-induced intestinal barrier dysfunction by modulating AMPK and NF-κB through TLR2 (57). Andrographolide strengthens intestinal barrier function and boosts the abundance of *Akkermansia muciniphila*, exerting antihyperglycemic effects (58). *Akkermansia muciniphila* colonization alleviates jejunal mucosal barrier disruption caused by high fructose and stress (59). These findings underscore the significant contribution of *Akkermansia muciniphila* to gut barrier maintenance and its potential therapeutic value in gut-related disorders.

Mucin, a critical component of the gut’s protective mucus layer, plays a central role in the ecology and function of *Akkermansia muciniphila*. Recent studies have elucidated how *Akkermansia muciniphila*’s specialized mucin degradation enzymes enable it to colonize and thrive within this niche (60–63). Binding specificity to O-glycans on mucin is key to its adhesion and utilization (64). The bacterium’s adaptation to high mucin environments, through energy homeostasis regulation, showcases its metabolic flexibility (65). Berberine indirectly promotes *Akkermansia muciniphila* growth by stimulating gut mucin secretion, highlighting the importance of diet-microbiome interactions (66). A deficiency in *Akkermansia muciniphila* and mucin depletion are linked to intestinal barrier dysfunction and inflammation (67), while excessive mucin consumption can lead to barrier damage (10). Mucin acts as a functional niche, significantly impacting gut microbiota composition and functionality, and its depletion can have detrimental effects beyond mere *Akkermansia muciniphila* supplementation (28). These findings underscore the pivotal role of mucin in gut health and highlight *Akkermansia muciniphila*’s potential as a therapeutic target for gut-related disorders.

### *Akkermansia muciniphila* contributes significantly to the anti-inflammatory response

*Akkermansia muciniphila* has been identified as a significant contributor to the modulation of the host’s immune response. This Gram-negative anaerobe is known to interact with the host’s mucus layer, promoting gut barrier integrity and influencing the balance of the gut microbiota. Recent studies have highlighted *Akkermansia muciniphila*’s ability to reduce inflammation in various disease models. For instance, it has been demonstrated to decrease *Porphyromonas gingivalis*-induced inflammation and periodontal bone destruction, suggesting a protective role in periodontal disease (68). Furthermore, the tripeptide derived from *Akkermansia muciniphila* has been reported to mitigate sepsis, inflammation, and mortality, highlighting its broad anti-inflammatory effects (69).

*Akkermansia muciniphila*, a beneficial gut bacterium, exerts its effects on immune homeostasis and inflammation by influencing Toll-like receptors (TLRs) signaling. This bacterium can directly engage TLRs, such as TLR2 and TLR4, to regulate the activation of immune cells and the release of cytokines (70, 71). Its phospholipid components have been demonstrated to possess immunomodulatory properties, which can dampen inflammation and promote immune balance (72, 73). Additionally, Amuc_1100 activates TLR2 signaling, enhancing the intestinal barrier function and modulating the gut microbiota (74, 75). This protein can also influence the production of serotonin, thereby affecting mood and behavior (76). *Akkermansia muciniphila* protects against nonalcoholic steatohepatitis by modulating macrophage polarization and TLR2-activated γδT17 cells (77) and improves cognitive function in aged mice (78). These effects are partly mediated through the regulation of TLR signaling pathways. Furthermore, *Akkermansia muciniphila* can alleviate the effects of high-fat diet-induced obesity and insulin resistance by modulating gut TLR signaling (79). Its extracellular vesicles have been shown to modulate TLRs and tight junctions, thereby improving intestinal barrier function (80). These findings underscore the importance of *Akkermansia muciniphila* in maintaining immune balance and health through its interactions with TLRs.

### *Akkermansia muciniphila*: a potential keystone in managing intestinal inflammation

High-frequency word analysis reveals that *Akkermansia muciniphila* is strongly associated with intestinal inflammation. *Akkermansia muciniphila* has been implicated in both protective and pathogenic effects in inflammatory bowel disease (IBD). In mice with dextran sulfate sodium (DSS)-induced colitis, administration of *Akkermansia* has been shown to ameliorate colitis symptoms, suggesting a beneficial role in IBD (81). However, in gnotobiotic interleukin-10-deficient mice, the *Akkermansia muciniphila* strain ATCC BAA-835 does not promote short-term intestinal inflammation, indicating that its effects may be strain-specific (82). In experimental models of colitis, *Akkermansia muciniphila* has been shown to reduce peritonitis and improve intestinal tissue wound healing after a colonic transmural defect through a MyD88-dependent mechanism (83). Additionally, the bacterium’s outer membrane protein Amuc_1100 has been identified as regulating tryptophan metabolism in colitis, highlighting its potential as a therapeutic target (84). *Akkermansia* may protect against colitis-associated tumorigenesis by modulating CD8^+^ T cells (48). Clinical studies have also further indicated the potential of *Akkermansia muciniphila* as a therapeutic agent. In an open-labeled study, an increased relative abundance of *Akkermansia muciniphila* was associated with improvement in abdominal pain in irritable bowel syndrome patients (85). These findings suggest that *Akkermansia muciniphila* could be a valuable target for the development of new treatments for intestinal disorders.

### *Akkermansia muciniphila*: a pivotal regulator of metabolic diseases

High-frequency word analysis reveals that research on *Akkermansia muciniphila* in metabolic disorders has primarily concentrated on metabolic dysregulation and obesity. *Akkermansia muciniphila*, a mucin-degrading bacterium with a growing reputation, has emerged as a key player in the regulation of metabolic diseases (86). The following review highlights recent studies that have elucidated its role in obesity, type 2 diabetes, and related disorders. *Akkermansia muciniphila* has been shown to play a role in the improvement of metabolic profiles by reducing inflammation in chow diet-fed mice (87). A study by Shen et al. (88) reported that capsaicin’s anti-obesity effect in mice fed a high-fat diet was associated with an increase in *Akkermansia muciniphila* population. In humans, higher stool salt levels were associated with obesity and *Akkermansia muciniphila* depletion (89). *Akkermansia muciniphila* cross-talk with the intestinal epithelium controlled diet-induced obesity (90). The bacterium was found to be lower in severe obesity (91) and to be inversely correlated with the onset of inflammation and metabolic disorders during obesity in mice (92). In a study using NOD mice, Hansen et al. found that early-life treatment with vancomycin propagated *Akkermansia muciniphila* and reduced diabetes incidence (4). *Akkermansia muciniphila* has been found to be negatively correlated with hemoglobin A1c in refractory diabetes (93). A decreased abundance of *Akkermansia muciniphila* led to impaired insulin secretion and glucose homeostasis in lean individuals with type 2 diabetes (49). In a randomized, double-blind, placebo-controlled trial, Roshanravan et al. (94) found that sodium butyrate and inulin supplementation affected angiotensin signaling by promoting of *Akkermansia muciniphila* abundance in type 2 diabetes. Furthermore, it has been demonstrated that *Akkermansia muciniphila* can effectively mitigate metabolic dysfunction-associated fatty liver disease by regulating L-aspartate metabolism through the gut-liver axis (95). The *Ophiopogon japonicus* polysaccharide MDG has also been shown to inhibit non-alcoholic fatty liver disease by modulating the abundance of *Akkermansia muciniphila* (96). Additionally, *Akkermansia muciniphila* has been found to reduce fat accumulation by activating the nhr-49-mediated nuclear hormone signaling pathway in *Caenorhabditis elegans* (97). These findings collectively suggest that *Akkermansia muciniphila* represents a promising therapeutic target for the treatment of metabolic syndrome and related disorders.

### *Akkermansia muciniphila* and the gut-brain axis: insights into neuroprotective and cognitive benefits

High-frequency word analysis reveals that *Akkermansia muciniphila* is strongly associated with the gut-brain axis. *Akkermansia muciniphila* is increasingly recognized for its pivotal role in modulating the gut-brain axis, impacting various neurological conditions. Research has demonstrated that *Akkermansia muciniphila* ameliorates depression-like behaviors in mice exposed to chronic stress (45, 98), with its outer membrane protein, Amuc_1100, specifically alleviating stress-induced anxiety and depression (44). Notably, this probiotic strain also improves depressive symptoms by regulating 5-HT neurotransmitter levels in both the gut and brain (99), and its small extracellular vesicles suppress microglial activation, thereby attenuating cognitive dysfunction post-surgery (74). The benefits of *Akkermansia muciniphila* extend to Alzheimer’s disease, where it mitigates cognitive deficits and amyloid pathology in mouse models (100), Supplementation with *Akkermansia muciniphila* can prevent cognitive impairment in sleep-deprived mice (101). Moreover, it reverses cognitive impairment associated with high-fat diets in rats (102), and its abundance correlates with reduced anxiety and depression in socially defeated mice (103). The diversity of *Akkermansia muciniphila* in the gut microbiota is linked to Parkinson’s disease dementia (104), and its presence in the gut microbiota, as modulated by metformin, improves cognitive function in aged mice (105). These findings reinforce the critical role of *Akkermansia muciniphila* in maintaining neurological health, offering potential therapeutic avenues for cognitive and neurodegenerative disorders. The emerging evidence on *Akkermansia muciniphila*’s influence on the gut-brain axis underscores its potential as a therapeutic target for neurological conditions.

### Limitation and strength

*Akkermansia muciniphila* has garnered acclaim as a prospective probiotic, a notion largely substantiated by studies in animal models. Our bibliometric analysis, however, predominantly highlighted associations between *Akkermansia muciniphila* and inflammatory conditions and metabolic disorders, with less pronounced evidence regarding its relevance to other diseases. This discrepancy may stem from several factors: In many disease cohorts, the abundance of *Akkermansia muciniphila* does not demonstrate statistically significant fluctuations, implying minimal impact or involvement in certain pathologies. The probiotic benefits of *Akkermansia muciniphila* in contexts beyond metabolism and inflammation remain underexplored, potentially due to a scarcity of targeted functional assays evaluating its activity in diverse disease states. Comprehensive clinical assessments featuring large patient populations are lacking, hindering definitive conclusions about *Akkermansia muciniphila*’s widespread efficacy and safety profile. Notably, some studies illustrate a close association between elevated *Akkermansia muciniphila* levels and disease onset, underscoring the complexity of its role in health and disease. For *Akkermansia muciniphila* to genuinely transition into a mainstream health-promoting organism, extensive large-scale clinical trials are warranted, coupled with vigilant monitoring for potential adverse effects. Such endeavors will provide critical insights necessary for establishing *Akkermansia muciniphila*’s utility and safety across various health spectra.

## Conclusion

*Akkermansia muciniphila* emerges as a cornerstone probiotic species, demonstrating potent anti-inflammatory properties by fortifying the gut barrier, generating short-chain fatty acids (SCFAs), and orchestrating immunological responses. Its potential in mitigating obesity is evidenced by improvements in insulin sensitivity, adiposity reduction, and body weight regulation. *Akkermansia muciniphila* fosters gut health through diverse strategies, encompassing barrier optimization, modulation of energy metabolism, facilitation of GLP-1 secretion, and biosynthesis of advantageous metabolites, which synergistically support weight management and metabolic homeostasis. By leveraging mucin utilization, competitive exclusion of detrimental microbes, promotion of commensal bacteria, and production of SCFAs alongside additional bioactive compounds, *Akkermansia muciniphila* plays a pivotal role in preserving gut health and augmenting overall welfare, thereby affecting neurological and metabolic disorders. Prospectively, *Akkermansia muciniphila*’s anti-inflammatory and gut-shielding characteristics position it at the forefront of therapeutic innovation. It holds promise in ameliorating inflammation-associated conditions, including malignancies and cardiovascular ailments. These hypotheses require validation through large-scale clinical cohort investigations to ascertain its efficacy. Concurrently, mechanistic insights can be sought through cell-based and animal model experiments to unravel underlying molecular interactions. Collectively, such endeavors will solidify *Akkermansia muciniphila*’s standing as a novel therapeutic candidate for a wider array of health challenges, underpinning its transformative potential in biomedical research and healthcare delivery.

## Data Availability

The original contributions presented in the study are included in the article/Supplementary material, further inquiries can be directed to the corresponding author.

## Supplementary material

SUPPLEMENTARY FIGURE S1 International cooperation information analyzed using PubMed and Scopus databases.
