# Supplementary figures and images for: The role of Akkermansia muciniphila in maintaining health: a bibliometric study
Source: Front Med (Lausanne). 2025 Feb 3;12:1484656. doi: 10.3389/fmed.2025.1484656 (PMC11833336; doi:10.3389/fmed.2025.1484656)

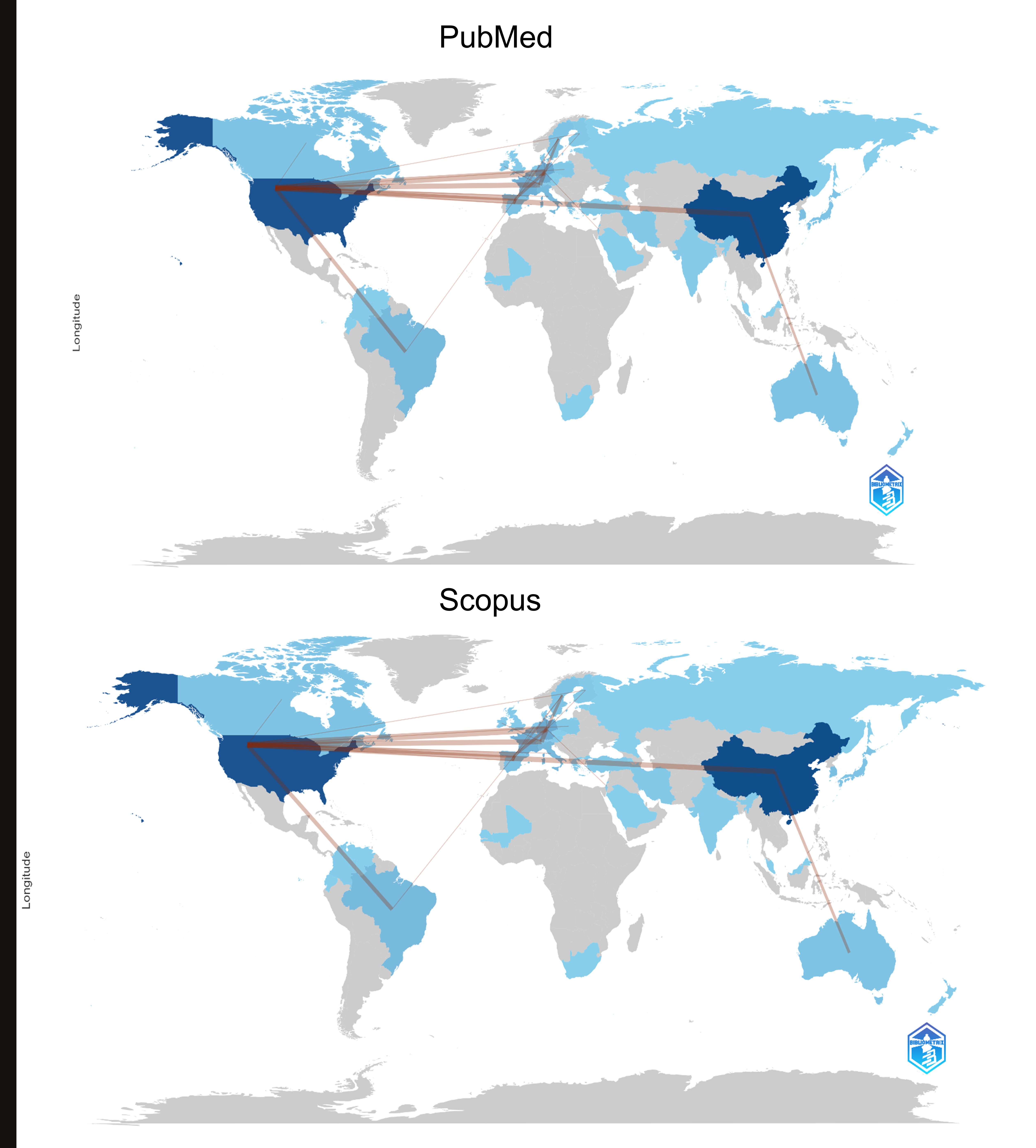

Supplement: SUPPLEMENTARY FIGURE S1 — International cooperation information analyzed using PubMed and Scopus databases. [file Image_1.TIF]
